# Management of symptomatic florid cemento-osseous dysplasia: Literature review and a case report

**DOI:** 10.4317/jced.54577

**Published:** 2018-03-01

**Authors:** Riccardo Aiuto, Federico Gucciardino, Roberta Rapetti, Sandro Siervo, Andrea-Edoardo Bianch

**Affiliations:** 1 Department of Periodontology and Oral Implantology – Istituto Stomatologico Italiano; 2 Department of Maxillofacial Surgery – Istituto Stomatologico Italiano; 3 Chief of Department of Periodontology and Oral Implantology – Istituto Stomatologico Italiano

## Abstract

**Introduction:**

Cemento-osseous dysplasia is a jaw disorder characterized by a reactive process in which normal bone is replaced by connective tissue matrix. There are different Cemento-osseous dysplasia entities. The treatment of these lesions, once diagnosed by radiology, is not required because generally they are asymptomatic. The localization is in the tooth-bearing areas of the jaws and its distribution is symmetric.

**Case Reports:**

In this case report, a 57-year-old Caucasian female patient was referred to our attention complaining of painful inflammatory events localized in the right angle of the jaw. The radiographic appearance, the distribution of several lesions and the positive vitality test of the involved teeth, supported the diagnosis of Florid Cemento-osseous dysplasia. Because of the symptomatology, the patient was submitted to surgery and the lesion and the second inferior right molar were removed. The histological examination of the specimens confirmed the diagnosis.

**Discussion:**

Many lesions that may exhibit a similar sclerotic appearance on conventional radiographs have to be differentiated and dental imaging can be used to discriminate between Florid COD and other lesions. Diagnosis of Florid Cemento-osseous dysplasia can be made with accurate clinical and radiographic assessment. In asymptomatic cases no treatment is required and the patient should have regular follow-up, but in this symptomatic case it was necessary to proceed with surgical intervention. The surgery treatment in the symptomatic case had a favourable prognosis and the two years follow-up has shown a complete healing. Given the abow, it is concluded that the choice of treatment must be selective according to the disease sites.

** Key words:**Cemento-ossifying dysplasia, fibro-osseous lesions, florid cemento-osseous dysplasia, cementoma.

## Introduction

Fibro-osseous lesions of the jaws are of particular interest to oral and maxillofacial surgeons, radiologists and pathologists, as they emphasize the crucial role of these specialists in the diagnostic process and therapy. The differential diagnosis is important because misdiagnosis may lead to unnecessary endodontic treatment, incisional biopsy or surgical removal. The term fibro-osseous lesion (FOL) is a generic designation of a group of jaw disorders (1,2).

In these lesions, bone is replaced by benign connective tissue matrix that displays varying degrees of mineralization in the form of woven bone or of cementum-like round acellular intensely basophilic structures indistinguishable from cementicles (3,4). The term FOL in the maxillofacial region is applied to cemento-osseus dysplasia (COD), fibrous dysplasia (FD) (5) and cemento-ossifying fibroma (COF) (6) and their subtypes (7).

COD represents a reactive process in which normal bone is replaced by a poorly cellularized cementum-like material and cellular fibrous connective tissue. They are classified, depending on clinical and radiographic findings, into three subtypes: periapical (limited to the anterior mandible), focal (single lesion), and florid (8).

These lesions, that present an atypical radiographic appearance, require a detailed clinical and laboratory workup to arrive at a correct diagnosis in order to develop an appropriate treatment (9,10).

On radiographic evaluation, during the early stage, a COD lesion may be detected as a round or oval apical radiolucency with a well-defined radiopaque border. In the second mixed stage, a radiolucent lesion may include radiopacities. In the mature stage, the internal mixed area becomes completely radiopaque with a thin radiolucent periphery (11).

Commonly, no treatment is required and only regular follow-up examinations are recommended (12,13).

In the 2005 edition, the World Health Organization (WHO) classification of odontogenic tumors, classified osseous dysplasia into four groups based on the location (14).

•Periapical Osseous Dysplasia (PCD)

•Focal Osseous Dysplasia (FocalCOD)

•Florid Osseous Dysplasia( is a widespread form of periapical osseous dysplasia, FOC)

•Familial Gigantiform Cementoma 

The term “florid” related to cemento-osseous dysplasia was introduced to describe the extensive manifestations of the disease in multiple quadrants of tooth-bearing area of the jaws (15,16). The populations most affected by this benign lesion are middle aged black women (17). FCOD lesions have a tendency towards bilateral, often quite symmetrical, location, and it is not unusual to find extensive lesions in all 4 posterior quadrants of the jaw (18,19).

FCOD is usually asymptomatic and diagnosed accidentally at routine dental radiographic examination. Dull pain, drainage, exposure of the lesion in the oral cavity, focal expansion and facial deformities are present when infection occurs (20,21). Radiographic image of FCOD shows lobular radiopacities that grow with the maturation of the lesion surrounded with radiolucent area and located mostly in mandibular premolar-molar region (22). This image is named “cotton wool” appearance and it is also seen in Paget’s disease (23).

## Case Reports

In 2007, a 49-year-old Caucasian woman was referred to our ward for a routine dental check up. Her past medical history was not notable, and there was no evidence of systemic disease.

On intraoral examination, the patient was asymptomatic, there was no swelling of the oral mucosa and all present teeth were vital except the second upper left molar. A panoramic X-Ray showed different apical lesions. In the jaw the apical area of incisors and the edentulous area of the right third molar were involved. In this latter area, the lesion extended mesiodistally from the distal root of the second molar and from the crest of the edentulous alveolar ridge to the inferior alveolar canal. The lesion had a somewhat multilocular appearence, a radiolucent area surrounded radiopaque structures, and the bone adjoining the borders of the lesions was sclerosed, giving a pseudo-corticated appearance. There was no root resorption or fusion of the lesions to the involved teeth. In the maxilla we could observe radiolucent lesions apically at the second right incisor and at the first left premolar. Because of the asymptomatic nature of the lesions, no further treatments were proposed. The patient was advised to have a routine follow-up examination six-months later, but she returned only in 2015 complaining of recurrent episodes of abscesses and exhibiting a significant degree of mobility of the second right molar. During this period the patient did not attend regular recall visits a tour department and she underwent endodontic treatment alio loco. She recalled the use of antibiotics without specifying type and duration.

**Figure 1:**
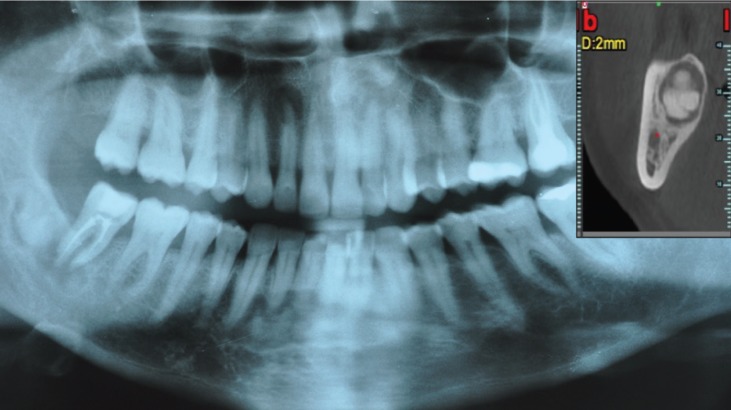
Orthopantomography 2014.

A new panoramic radiograph (Fig. 1) showed an enhancement of the diameter of the radiolucent lesions, which were disposed apically to the second upper right incisor and to the first upper left premolar. The lesion involving the lower incisors appeared essentially unchanged. In the right posterior area of the mandible the radiolucent lesion that surrounded radiopaque structures was substantially the same, but with enhanced dimensions in apico-coronally extension (the inferior alveolar canal appeared involved) while apically to the second right molar a new radiolucent area appeared.

On March 2014 a CT examination was performed in both jaws. In the vertical reconstructions of the maxilla multiple patchy well defined hyperdense lesions with hypodense rim located around root apices of both second incisors and the first left premolar were present. A sclerotic margin surrounded these lesions. Lesions above both lateral incisors were connected with buccal cortical bone without interruption. In the axial view we could observe the lesion that surrounding the first left premolar extended to the lingual cortical, no interruption. Panoramic-like reconstructions demonstrated the symmetry and the mesio-distal extension of these lesions. There were no root resorptions in any case. In the mandible multiple patchy well defined hyperdense lesions with hypodense rim and sclerotic borders located around root apices of the lower incisors were present, similar to the upper lesions in the maxilla. These lesions were in contact with the lingual cortical plate with no interruption of it. In the edentulous area of the right third molar the lesion appeared amorphous, lobulated and mixed radiolucent and radiopaque masses (cotton-wool appearance) with sclerotic borders. In the axial view the lesion looked something like an impacted tooth. Inferiorly to the second molar another radiolucent lesion that eroded the buccal cortical plate was present. The panoramic view showed that there were not interruptions of the inferior alveolar canal.

FCOD lesions that are not infected do not require any treatment procedure, but because of the septic complication and the symptomatology, a decision was made to extract this molar, to enucleate the lesion and to do histological examination. After the completion of the clinical and radiographic examination, the patient was scheduled for surgery under general anesthesia.

**Figure 2:**
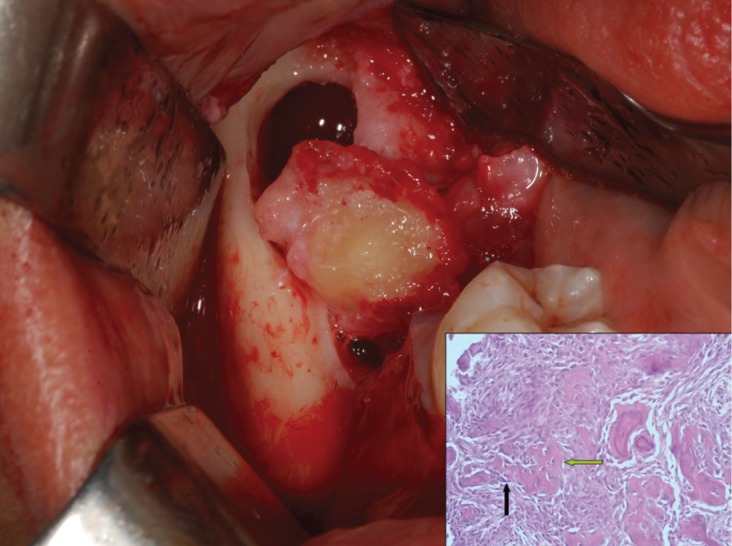
Removal of the lesion.

During surgery, a intrasulcular buccal incision of the second right mandibular molar was performed with a mesial extension to the distal side of the first right mandibular molar and a mucoperiosteal flap was raised. After the extraction of the second right inferior molar, the apical lesion was enucleated (Fig. 2). This lesion seemed to be inflammatory connective tissue. The lesion appeared bicameral, in fact, distal to the lesion previously described, a block section of a hard-elastic tissue, surrounding a dense calcified structure has been removed. After an accurate toilette of the cavity, clean cortical bony walls were observed. The affected area was irrigated with saline solution and local antibiotic (Rifocin). The flap was sutured with interrupted sutures. The patient was dismissed with routine postoperative instructions (antiseptic rinses) and antibiotic therapy: 1g amoxicillin/12h during 10 days.

**Figure 3:**
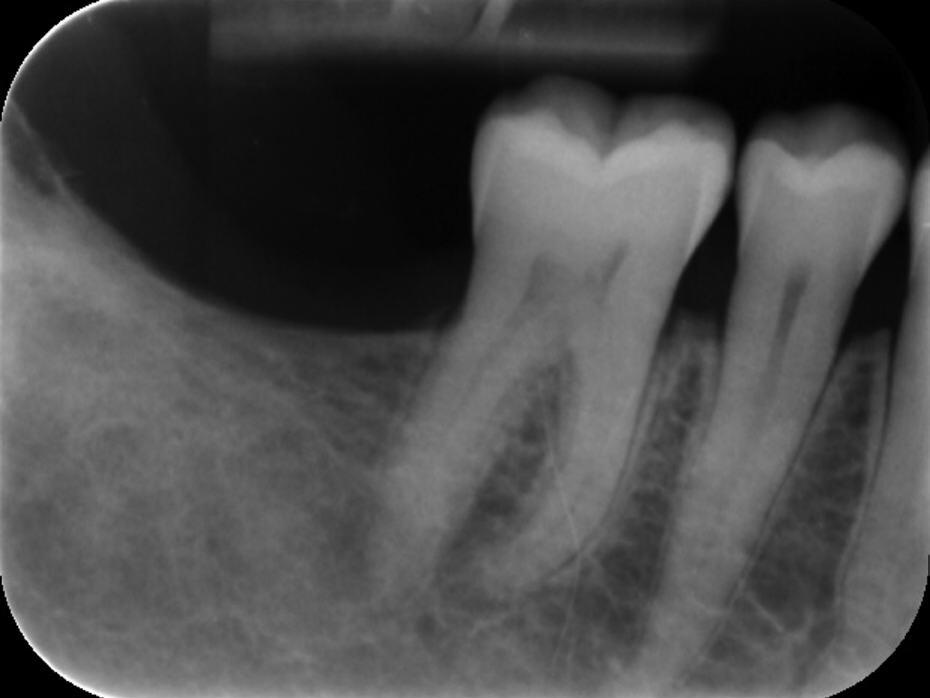
Two years follow-up.

The patient presented for radiography control 6 months after surgery. The patient was called back to our department 2 years after surgical treatment for a new radiographic follow-up that shows a complete healing (Fig. 3).

## Discussion

Our diagnostic hypotheses, based on medical history, clinical and radiological information was Florid Cemento-Osseous Dysplasia. Histological results show “ginger root” bony trabeculae surrounded by connective tissue matrix highly cellularized. There are bony trabeculae surrounded by activated osteoblast and osteoclast. At this point, diagnosis is made with clinical-pathological relationships:

•Focal cemento-osseus dysplasia is present if the posterior quadrant is involved

•Periapical cemento-osseus dysplasia is present if it involves an anterior quadrant 

•Florid cemento-osseus dysplasia is present if it occurs in more quadrants

All of these clinical situations share the same histological features including cemento-ossifyng fibroma from which they only differ by the lack of a capsule.

Etiology of cemento-osseus dysplasia and cemento-ossifying fibroma is not yet clearly understood, but the close proximity to the periodontal ligament may lead to the hypothesis that both cemento-osseus dysplasia and cemento ossifying fibroma originate from the same tissue. Furtherly, cemento-osseus dysplasia may represent a reactive lesion, whereas fibroma looks like a neoplasm. The former is developed apically to the root or on a site of tooth extraction , whereas COF often did not exhibit association with teeth but causes root displacement without root resorption.

Radiologically, the radioopaque aspect in Focal Cod is irregular or diffuse like “cotton wool”, otherwise radiodensity in COF is smoothly distributed like “snow flakes”. The explanation is the histopathological exam, “cotton wool” represents “ginger root” bony trabeculae in Focal COD, in contrast “snowflakes” appearance may reflect the bony trabeculae seen in COF (24). Regardless of radiologic features, we had more quadrants involved, so we were thinking about a florid COD. It’s important to establish a correct diagnosis because in an asymptomatic patient there is no need for treatment, and a regular follow-up is enough. Many lesions that may exhibit a similar sclerotic appearance on conventional radiographs have to be differentiated and dental imaging can be used to discriminate between Florid COD and other lesions.

In the presented case radiological lesions show a “cotton wool” aspect which is also typical of Paget’s disease, but the latter usually affects the entire mandible in addition with a loss of the lamina dura (whereas here lesions are well localized apically to teeth and lamina dura is not involved). Paget’s disease often involves other bones such as the spine or femur and causes biochemical serum changes as elevated alkaline phosphatase levels, which on the other hand is within normal limits in patients with FOCD (8,25).

A further radiopaque lesion that appears with even greater frequency is idiopathic osteosclerosis (IOS) but it has no radiolucent periphery, that otherwise is present in COFs, FD, odontomas and in our case and even it causes no expansion (26,27). Moreover IOS lacks the typical symmetry that is present generally in Florid COD. In this case the lesion had no alveolar bone expansion and the vitality of the involved tooth supported the diagnosis of FCOD (8). In the radiolucent-radiopaque mixed stage and the radiopaque stage, the differential diagnosis might include odontoma, cementoblastoma, osteoblastoma and ameloblastoma. The complex form of an odontoma can display all the radiological features of Cemento ossifyng fibroma (COF). Although the majority of odontomas do not exceed the dimensions of normal teeth, a number of very large odontomas, especially in the complex form, have been reported (28). Cementoblastoma characteristically is fused to the root (29), involves posterior mandibular segments and lesions are solitary. Often teeth have decreased sensitivity or are not vital, but in COD generally they remain vital. Osteoblastoma, as well as osteoid osteoma, causes dull, nocturnal pain. These symptoms lack in FCOD (8), so these two lesions can be eliminated from the list of the differential diagnosis.

Ameloblastoma is a benign tumor. It originates from odontogenic epithelium and is characterized by painless slow-growing swelling. Generally it occurs in the mandible, especially in the posterior area and ascending ramus, producing root resorption. We have not seen these characteristics in our case.

About FCOD management, it is reasonable to keep asymptomatic patient under observation. In the absence of clinical signs, it would be recommended a revaluation with panoramic radiograph every 2 or 3 years. When the patient is symptomatic the management becomes more difficult because chronic inflammation and infection develop within densely mineralized tissue. In this case, antibiotics have poor tissue diffusion and generally are not effective. Therefore, biopsy increases the risk of infection and it’s not reasonable to remove these lesions in asymptomatic patients (26,30).

Florid Cemento-osseous dysplasia needs an accurate clinical and radiographic assessment to be diagnosed. In the asymptomatic cases treatment is unnecessary, the patient should have a regular follow-up to check any changes. However, in the aforementioned case, since it was symptomatic, and considering that it was evolving in a chronic infection due to diseases of the adjacent teeth, it has been necessary to do surgery to solve the algic dysfunctional condition. Infact, the radical surgery treatment in the symptomatic cases has a favourable prognosis and allows a complete healing. The choice of this treatment should be selective according to the disease sites. The goal of this work is to highlight that symptomatic cases of bacterial FCOD overlap of maxillary bones, can not be solved with drug therapy alone, radical surgery leads to a recovered fully demonstrates clinically and with radiology.
